# Supplementary material for: Generation of Infectious Prions Amenable to Site-Specific Click Chemistry
Source: ACS Chem Biol. 2026 Jun 9;21(6):1525–32. doi: 10.1021/acschembio.6c00288 (PMC13288451; doi:10.1021/acschembio.6c00288)
Supplement: Supplementary file 1 [file cb6c00288_si_001.pdf]

Supplemental Methods and Figures

Generation of Infectious Prions Amenable to Site-specific Click Chemistry

Ryan G. Campbell[†,1], Jolene N. Iseler[†,1], Abigail M. Schwind[1], and Surachai Supattapone[1,2,*]

[†]These authors contributed equally to this work

Departments of Biochemistry and Cell Biology[1] and Medicine[2], Geisel School of Medicine at Dartmouth, Hanover, New Hampshire 03755, USA

Protease Digestion of Recombinant PrP^{Sc} samples:

To detect the presence of protease-resistant PrP^{Sc}, samples were subjected to Proteinase K (PK) digestion and analyzed by western blotting, as described in Walsh *et al*. 2023[1]. In brief, samples (50 µL) were incubated with 20 µg/mL of PK for 30 min at 37℃ at 750 rpm in a 3 mm orbit Ohaus shaker (Parsippany, NJ).

Preparation and Protease Digestion of Brain Homogenates:

Brain derived samples were all prepared as 10% (w/v) in PBS without calcium or magnesium and underwent bead mill homogenization and centrifugation at 200 x *g* for 30 sec to remove brain debris and to isolate the PrP^{Sc}-containing supernatant for PK digestion. 50 µL of 10% (w/v) each brain homogenate was diluted with 50 µL of 1X PBS with 1% Triton X-100, containing either 100 µg/mL PK (+PK samples) or water (-PK samples).

PrP^{Sc} Visualization:

After PK digestion, samples were quenched with 4 mM phenylmethylsulfonyl fluoride (PMSF), boiled in Laemmli SDS loading buffer (Bioland Scientific, Paramount, CA) with 2-Mercaptoethanol for 10 min at 95°C and run on a 12% polyacrylamide gel. Protein was transferred to a polyvinylidene fluoride (PVDF) membrane and was blotted as described previously with mAb 27/33 (epitope: 142-149, mouse numbering) and horseradish peroxidase-linked sheep anti-mouse antibodies[2]. Fluorophore conjugated PrP^{Sc} was imaged after SDS-PAGE using an Azure 600 bioimager (Azure Biosystems, Dublin, CA). The following excitation and emission settings were used to acquire images for each fluorophore: AF647, Ex. 628/16 nm, Em. 684/12 nm; BODIPY, Ex. 472/15 nm, Em. 513/8.5 nm; Cy 7.5, Ex. 784 nm, Em. 832/18.5 nm.

Preparation of Inoculum:

PrP^{Sc} propagation reactions were washed of unconverted PrP^{C} by centrifuging for 30 min at 18,000 x *g* and aspirating the supernatant. After washing, inoculum was diluted 1:10 in 1X PBS + 1% bovine serum albumin. *In vivo* inoculation was performed as described in Piro *et al.* 2009[2]. For Cy7.5-PrP^{Sc} injection, protein was concentrated to 8.9 µM and a total volume of 15 µL was injected intracerebrally, divided between two sites.

Scrapie Inoculation and Diagnosis:

Knock-in female mice expressing bank vole M109 PrP (termed kiBVM mice)[3] between 4-5 weeks old were intracerebrally injected with 30 µL inoculum at 0.6 µg/mL PrP. Scrapie symptoms were monitored daily and a clinical diagnosis of scrapie was made based on the onset of wide gait, shaking, and/or circling[4]. Data was analyzed using GraphPad Prism 10; time to onset was reported as mean and standard error of the mean (SEM).

Neuropathology:

Within 24 hrs of scrapie symptom onset, sick mice were sacrificed and whole mouse brains were harvested and sliced in half parasagittally. For Western blot samples, ½ brains were frozen at -80℃ until western blot sample preparation[2]. For pathology samples, ½ brains were placed into 10% formalin for fixation, decontaminated by immersion in 88% formic acid for 1 hr, paraffin embedded, sliced parasagittally, and stained with hematoxylin and eosin by the Dartmouth Hitchcock Research Pathology Service Core (Lebanon, NH). Tissue samples were scored between 0-5 as previously described[5].

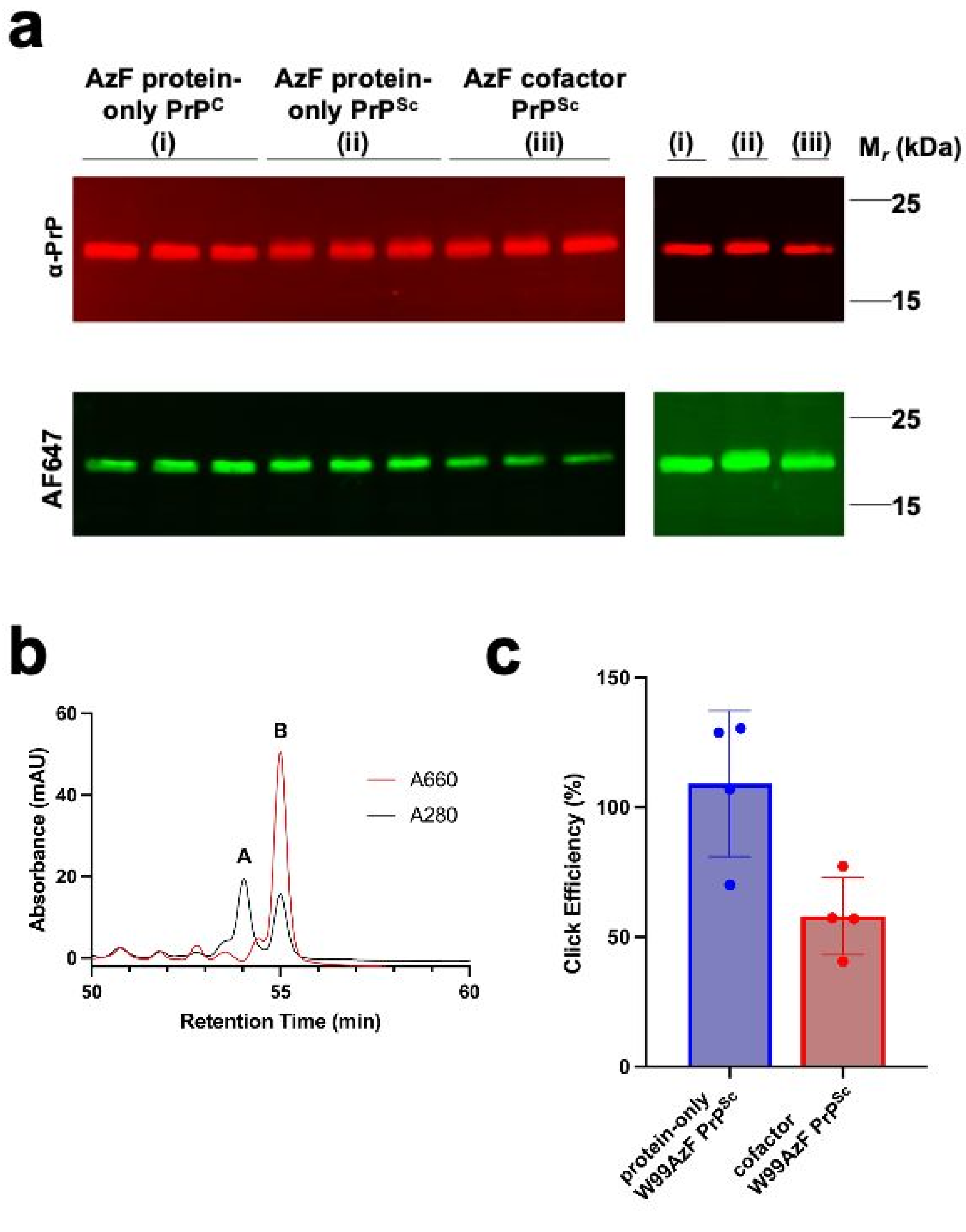

Figure S1: Determination of Click Efficiency of Protein-only and Cofactor AzF PrP^{Sc}.

(A) 3 µg of either AzF PrP^{C} (i), AzF protein-only PrP^{Sc} (ii), or AzF cofactor PrP^{Sc} (iii) was reacted with 20 µM AF647-DBCO for 1.5 hrs, 25°C, 750 rpm (Ohaus, Parsippany, NJ). The reaction was quenched with a final concentration of 100 mM sodium azide. Samples were boiled in 1X Laemmli SDS loading buffer (Bioland Scientific, Paramount, CA) with 2-Mercaptoethanol for 10 min at 95°C and run on a 12% polyacrylamide gel. Protein was transferred to a polyvinylidene fluoride (PVDF) membrane and was blotted as described previously with mAb 27/33 (epitope: 142-149, mouse numbering) and Amersham CyDye800 goat anti-mouse (Cytiva, Marlborough, MA) antibodies to quantify total PrP (Ex. 784 nm, Em. 832/18.5 nm). AF647-PrP conjugation was directly measured using AF647 fluorescence (Ex. 628/16 nm, Em. 684/12 nm) on an Azure 600 bioimager (Azure Biosystems, Dublin, CA). All samples are technical replicates, including the three samples run on a separate smaller gel (right panel).

(B) Quantification of click reaction efficiency for AzF PrP^{C} with AF647-DBCO using analytical HPLC. AF647-AzF PrP^{C} (14.4 µg) was prepared as described in the materials and methods, and the reaction was quenched with excess sodium azide. This clicked PrP^{C} was combined with unclicked AzF PrP^{C} (21.6 µg), such that the final concentration was 40% clicked PrP^{C}, 60% unclicked PrP^{C}. The protein sample was denatured in 2M urea and loaded onto a C4 reverse phase column (Sepax Technologies, Inc., Newark, DE). Reverse phase chromatography was performed as described in Makarava and Baskakov, 2008[6] to resolve clicked PrP^{C} from unclicked PrP^{C}. Both PrP species eluted at ~32% acetonitrile, typical for PrP[6]. Unclicked PrP (Peak A) was confirmed by 280nm absorbance and a clear lack of 660nm absorbance. AF647-AzF PrP (Peak B) was confirmed by its large 660nm absorbance, consistent with AF647 conjugation. After integrating peaks A and B, the total protein composition was 40.27% peak A, 59.73% peak B, which is as expected if AF647-DBCO clicked quantitatively to AzF PrP^{C} in the initial reaction to create the clicked-PrP peak B. The congruence between input protein ratio and the HPLC peak integrations suggests a quantitative reaction between AzF PrP^{C} and AF647-DBCO.

(C) The percent of total AzF PrP clicked to AF647-DBCO was determined by dividing the AF647 fluorescence (**A**, bottom panel) by total PrP signal (**A**, top panel). The AF647/CyDye800 ratio for AzF protein-only PrP^{Sc} and AzF cofactor PrP^{Sc} was normalized by setting the average AF647/CyDye800 ratio for AzF PrP^{C} to 100%, consistent with the click reaction for AzF PrP^{C} being quantitative. Normalized, technical quadruplicate data was plotted as mean and standard error of the mean using GraphPad Prism 10 (GraphPad, San Diego, CA).

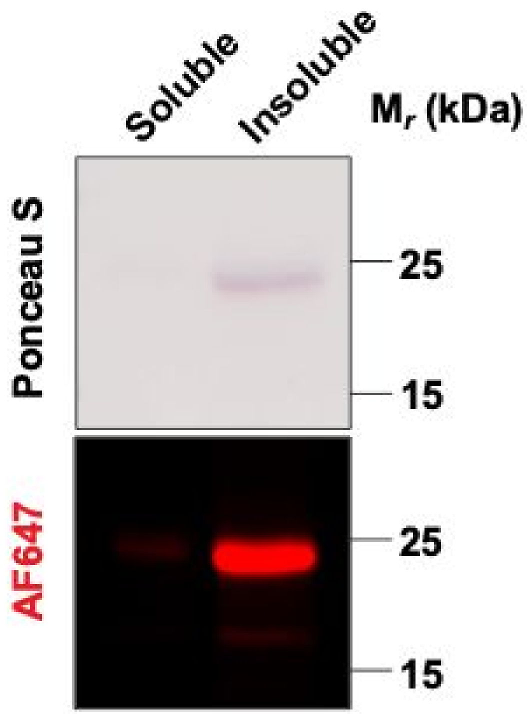

Figure S2: Insolubility of AF647-AzF PrP^{C}

Following HPLC purification, a single fraction of AzF PrP^{C} (100 μM) was reacted with AF647-DBCO (220 μM) in 100 mM Tris, pH 7.5, 25°C, 16 hrs, and then lyophilized. Protein was resuspended as previously described[1]. Centrifugation of AF647-AzF PrP^{C} at 18,000 xg for 20 min yielded a pellet of insoluble protein. The supernatant (1 mL) was collected and the pellet was washed once with 1X PBS and resuspended in 1 mL of 1X PBS. 50 μL samples of supernatant (soluble) and pellet (insoluble) were prepared and boiled in 1X Laemmli SDS loading buffer (Bioland Scientific, Paramount, CA) with 2-Mercaptoethanol for 10 min at 95°C and run on a 12% polyacrylamide gel. Fluorescent gel imaging using an Azure 600 bioimager (Azure Biosystems, Dublin, CA) indicated that AF647-AzF PrP had precipitated into the insoluble pellet fraction (Ex. 628/16 nm, Em. 684/12 nm). Protein was transferred to a polyvinylidene fluoride (PVDF) membrane. Subsequent total protein staining with Ponceau S indicated that most of the protein had precipitated.

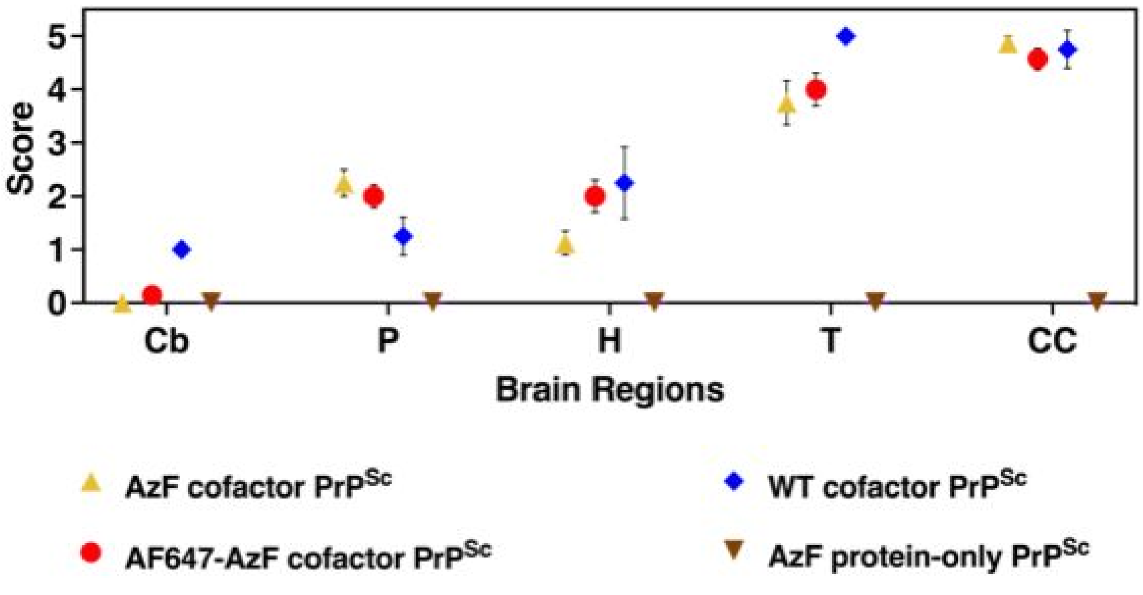

Figure S3: Neuropathology of Mice Inoculated with AzF PrP^{Sc} and AF647-AzF PrP^{Sc}
Representative hematoxylin and eosin (H&E) stained images showing vacuolation in the cerebral cortex of mice inoculated with AzF protein-only PrP^{Sc}, AzF cofactor PrP^{Sc}, or AF647-AzF cofactor PrP^{Sc}, as indicated.

References

(1) Walsh, D. J.; Schwind, A. M.; Noble, G. P.; Supattapone, S. Conformational diversity in purified prions produced in vitro. *PLoS Pathog* **2023**, *19* (1), e1011083. DOI: 10.1371/journal.ppat.1011083.
(2) Piro, J. R.; Harris, B. T.; Nishina, K.; Soto, C.; Morales, R.; Rees, J. R.; Supattapone, S. Prion protein glycosylation is not required for strain-specific neurotropism. *J Virol* **2009**, *83* (11), 5321-5328.
(3) Arshad, H.; Eid, S.; Mehra, S.; Williams, D.; Kaczmarczyk, L.; Stuart, E.; Jackson, W. S.; Schmitt-Ulms, G.; Watts, J. C. The brain interactome of a permissive prion replication substrate. *Neurobiology of disease* **2025**, *206*, 106802. DOI: 10.1016/j.nbd.2025.106802 From NLM Medline.
(4) Prusiner, S. B.; Cochran, S. P.; Groth, D. F.; Downey, D. E.; Bowman, K. A.; Martinez, H. M. Measurement of the scrapie agent using an incubation time interval assay. *Ann Neurol* **1982**, *11* (4), 353-358.
(5) Deleault, N. R.; Harris, B. T.; Rees, J. R.; Supattapone, S. Formation of native prions from minimal componenets in vitro. *Proc Natl Acad Sci U S A* **2007**, *104* (23), 9741-9746.
(6) Makarava, N.; Baskakov, I. V. Expression and purification of full-length recombinant PrP of high purity. *Methods Mol Biol* **2008**, *459*, 131-143.
